# A Sub-Element in PRE enhances nuclear export of intronless mRNAs by recruiting the TREX complex via ZC3H18

**DOI:** 10.1093/nar/gku350

**Published:** 2014-04-29

**Authors:** Binkai Chi, Ke Wang, Yanhua Du, Bin Gui, Xingya Chang, Lantian Wang, Jing Fan, She Chen, Xudong Wu, Guohui Li, Hong Cheng

**Affiliations:** 1 Shanghai Key Laboratory of Molecular Andrology, State Key Laboratory of Molecular Biology, Institute of Biochemistry and Cell Biology, Shanghai Institutes for Biological Sciences, Chinese Academy of Sciences, Shanghai 200031, China; 2 National Institute of Biological Sciences, 7 Science Park Road, Zhong Guan Cun Life Science Park, Beijing 102206, China; 3 Laboratory of Molecular Modeling and Design, State Key Laboratory of Molecular Reaction Dynamics, Dalian Institute of Chemical Physics, Chinese Academy of Sciences, Dalian 116023, China

## Abstract

Viral RNA elements that facilitate mRNA export are useful tools for identifying cellular RNA export factors. Here we show that hepatitis B virus post-transcriptional element (PRE) is one such element, and using PRE several new cellular mRNA export factors were identified. We found that PRE drastically enhances the cytoplasmic accumulation of cDNA transcripts independent of any viral protein. Systematic deletion analysis revealed the existence of a 116 nt functional Sub-Element of PRE (SEP1). The RNP that forms on the SEP1 RNA was affinity purified, in which TREX components as well as several other proteins were identified. TREX components and the SEP1-associating protein ZC3H18 are required for SEP1-mediated mRNA export. Significantly, ZC3H18 directly binds to the SEP1 RNA, interacts with TREX and is required for stable association of TREX with the SEP1-containing mRNA. Requirements for SEP1-mediated mRNA export are similar to those for splicing-dependent mRNA export. Consistent with these similarities, several SEP1-interacting proteins, including ZC3H18, ARS2, Acinus and Brr2, are required for efficient nuclear export of polyA RNAs. Together, our data indicate that SEP1 enhances mRNA export by recruiting TREX via ZC3H18. The new mRNA export factors that we identified might be involved in cap- and splicing-dependent TREX recruitment to cellular mRNAs.

## INTRODUCTION

mRNA export is a critical step in eukaryotic gene expression. It mainly occurs via specific interactions between the mRNA export receptor, known as TAP/P15, and the mRNA export adaptors, Aly and Thoc5, which are components of the TREX complex (TREX) (1,2). Except for Aly and Thoc5, the human TREX also contains UAP56/URH49 as well as the five other components of the six-subunit THO complex (Thoc1, Thoc2, Thoc5, Thoc6, Thoc7 and Tex1) (3,4). Both formation and function of TREX are conserved from yeast, drosophila to humans. Aly and UAP56/URH49 are conserved in all of these three species. Although the drosophila THO complex is highly similar to its human counterpart, the yeast THO complex is formed by five subunits, including Tho2, Hpr1, Tex1 as well as two proteins, Thp2 and Mft1, which do not have apparent human and drosophila homologues (3,5–7). In recent years, in addition to Aly, UAP56/URH49 and THO, increasing number of human TREX components have been reported, and these components also play important roles in mRNA export (8–10).

Prior to nuclear export, pre-mRNAs undergo multiple RNA processing steps. These steps include capping at the 5′ end, splicing to remove introns, and polyadenylation at the 3′ end. mRNA export is physically and functionally coupled to these processing steps probably via protein–protein interactions between mRNA export factors and components of mRNA processing machineries. This coupling on one hand maintains the high efficiency of mRNA export, and on the other hand may ensure that only fully processed mRNA can be exported to the cytoplasm for translation into proteins. In higher eukaryotes, most genes contain multiple introns, and TREX is recruited during a late step of splicing (3). Splicing significantly enhances TREX recruitment and mRNA export (3,11,12). The underlying mechanism most likely involves factors that are present in the spliceosome and/or spliced messenger ribonucleoprotein particle ( spliced mRNP) and function in recruitment/stabilization of TREX on the mRNA. However, these factors remain to be identified.

Studies on viral mRNA export have made important contributions to understanding the mechanisms for cellular RNA export. To maximize the production of viral proteins, several viruses have evolved proteins and/or *cis*-acting RNA elements that specifically recruit cellular mRNA export factors for exporting viral mRNAs. Efforts to identify the cellular targets of these viral proteins and RNA elements have led to the identification of Crm1 and TAP as essential human nuclear export factors (13–15). Hepatitis B virus (HBV) encodes several intronless viral mRNAs. Expression of viral intronless mRNAs that encode surface proteins is dependent upon an RNA element named post-transcriptional regulatory element (PRE) (16). PRE was initially discovered by two groups in the early 1990's (16–18). Subsequently, it was found that in the absence of PRE, viral mRNAs that encode surface proteins are degraded in the nucleus (17). Based on these data, it was proposed that PRE might function in nuclear export of these viral mRNAs (17). Consistent with this, PRE has been shown to promote the expression of cDNA transcripts in the absence of any viral protein (18). The function of PRE is not inhibited by the Crm1 inhibitor leptomycin B, indicating that PRE uses a Crm1-independent pathway (19). A previous study showed that overexpression of the VSV M protein blocks PRE-dependent protein expression but not tRNA export (19,20). These data indicate that the export pathway utilized by PRE is also distinct from that of tRNA. Finally, PRE is thought to use a different pathway from the Mason–Pfizer monkey virus CTE element, which enhances mRNA export by directly recruiting the cellular mRNA export receptor TAP (15,19,21). Thus, currently, the nuclear export pathway that is used by PRE remains unclear.

PRE functions in the absence of any viral proteins, indicating that some cellular protein(s) that are specifically recruited by PRE are important for its function (18). Studies to identify these cellular proteins led to the discovery of three proteins, glyceraldehyde-3-phosphate dehydrogenase (GAPDH), PTB and the La protein (22–24). Nevertheless, evidence for these proteins functioning in PRE-dependent mRNA export is still lacking, and the functional PRE-interacting proteins remain to be identified. Considering that PRE is able to functionally substitute for introns in supporting the efficient expression of cDNA transcripts, identification of these functional PRE-interacting proteins may provide insights into the mechanism(s) by which splicing facilitates mRNA export.

In this study, we found that PRE promotes the nuclear export of cDNA transcripts via specifically and efficiently recruiting TREX. Our data demonstrate that the zinc finger protein ZC3H18 directly binds to the SEP1 RNA (Sub-Element of PRE), interacts with the TREX component Thoc2 and functions in SEP1-mediated TREX recruitment and mRNA export. Among the SEP1-associating proteins, we identified multiple new cellular mRNA export factors, including ZC3H18, a component of the nuclear cap-binding complex (CBC) named ARS2, a component of the exon-junction complex (EJC) named Acinus, and the U5 snRNP component, Brr2.

## MATERIALS AND METHODS

### Plasmids and antibodies

To make the wG and cG constructs, the human β-globin gene with or without introns was inserted into the *Kpn*I and *Eco*RV site of pcDNA3. To construct the cG-PRE and cG-rPRE plasmids, the HBV PRE sequence (nucleotides 1239–1805 in the HBV genome) and its reverse complement sequence was amplified by polymerase chain reaction (PCR) and inserted to *Not*I and *Xba*I sites of the cG construct, respectively. To construct the truncated PRE plasmids, different PRE fragments were amplified by PCR and inserted into the *Not*I and *Xba*I sites of the cG construct. To construct the wS and cS plasmids, three exons of the *Xenopus laevis* Smad gene with or without AdML introns were amplified using PCR and inserted into the *Kpn*I-*Eco*RV site of pcDNA3. The PRE sequence was inserted into the *Not*I and *Xba*I sites of cS to make the cS-PRE construct. The SEP1 sequence (nucleotides 1590–1705) and its reverse complement sequence (rSEP1) were inserted into the *Hin*dIII and *Kpn*I sites of pcDNA3 plasmid to make the constructs used for *in vitro* transcription and RNP IP. For MS2-MBP purification, three MS2 binding site sequences were inserted into the 3′ of SEP1 and rSEP1 in pcDNA3 (*Eco*RI-*Xba*I site). To construct the GST-ZC3H18-zinc finger plasmid (GST-Z-ZD), nucleotides 1–792 were amplified and inserted into the *Eco*RI-*Xho*I sites of plasmid pGEX 6P1. To make the PRE construct for co-injection experiment, the PRE sequence was inserted into the *Hin*dIII and *Kpn*I sites of pcDNA3. The plasmid encoding tRNA (pSUPER-tRNA) and the MS2-MBP plasmid were described previously (12,25). Antibodies against Thoc2, Thoc5, UAP56, Aly, CBP80 and eIF4A3 were described previously (4). The negative control antibody used for IPs was against fSAP130. The GAPDH, tubulin, Flag and SC35 antibodies were purchased from Sigma. The Brr2 and ZC3H18 antibodies were purchased from Abnova.

### Cell culture and RNAi

HeLa cells were cultured in Dulbecco's modified Eagle's medium supplemented with 10% fetal bovine serum (Biochrom). For shRNA-mediated knockdown, pLKO.1 plasmid inserted with DNA oligonucleotide encoding shRNA, psPAX2 plasmid and pMD 2.G plasmid was introduced into 293 FT cells. The media containing viruses were harvested after 48 h and added to HeLa cells. After another 24 h, 2 μg/ml puromycin was added to the medium. Seventy-two hours later, cells were used for transfection. Cells were harvested for fluorescence *in situ* hybridization (FISH), western and RT-PCR analyses 24 h after transfection. The shRNAs targeting sequences are shown in Supplementary Table S1. To knock down UAP56/URH49, the UAP56 and URH49 siRNA described previously were used (26). The siRNAs targeting sequences are also shown in Supplementary Table S1.

### FISH and immunofluorescence

For FISH and immunofluorescence, HeLa cells were plated on fibronectin coated coverslip bottom of 35 mm dishes. To detect the RNAs transcribed by transfecting the globin or Smad reporter constructs, a high-performance liquid chromatography-purified Alexa 548 conjugated 70 nt probe that hybridizes to pcDNA3 vector sequence (vector probe) was used.To detect the Smad mRNA co-injected with tRNA or PRE, Smad probe that hybridizes to a region of Smad mRNA was used (26). HeLa cells were transfected with plasmids (1 μg) and fixed with 4% paraformaldehyde for 15 min 24 h post-transfection. Cells were washed with 1× phosphate buffered saline (PBS) for three times and permeabilized with 0.1% Triton in PBS for 15 min. Cells were washed with 50% formamide twice and incubated at 37°C with FISH probes for 16 h. Cells were then washed with 50% formamide in 1× saline-sodium citrate buffer (SSC) for four times, and images were captured with an EM-CCD camera on an inverted microscope (Olympus). To detect polyA RNAs, FISH was performed as previously described using a HPLC-purified Alexa 548 conjugated oligo dT (70) probe (26). To carry out immunofluorescence, 4% PFA fixed cells were incubated with the SC35 antibody 1:200 diluted in blocking buffer (1× PBS, 0.1% Triton, 2 mg/ml bovine serum albumin) for 30 min at the room temperature. Cells were then washed three times with PBS for 5 min each and incubated with the Alexa-488 labeled anti-mouse antibody 1:2000 diluted in blocking buffer for another 30 min at the room temperature, followed by DAPI staining and three washes in PBS for 10 min each. To quantify the FISH images, the area (*A*_W_), the average signal intensity of the whole cell (*I*_W_) of each transfected cell, the area (*A*_N_) and the average signal intensity of the nucleus (*I*_N_) of each transfected cell were determined using the Image J software. The background intensity (*I*_WB_ for whole cell, and *I*_NB_ for nucleus) was determined using an un-transfected cell, and the whole cell signal (W) was equal to *A*_W_ (*I*_W_−I_WB_). Similarly, the nuclear signal (N) was equal to A_N_ (*I*_N_−*I*_NB_), and the cytoplasmic signal (C) was equal to W−N. The ratio of the cytoplasmic/nuclear fluorescence signal (C/N ratio) was equal to (W−N)/N.

### RNP purification

^32^P labeled PRE-SEP1 and rSEP1 RNAs (2 μg) were incubated with 4 μg of purified MS2-MBP protein on ice for 30 min. Splicing dilution buffer (172 μl; 20 mM HEPES, pH 7.6 and 100 mM KCl) was added, and incubation was continued for another 20 min. The mixture was then added to 2.4 ml of standard splicing reaction and incubated at 30°C for 1 h. RNPs that assembled on the SEP1 and rSEP1 RNAs were isolated by gel filtration on a 1.5 cm/50 cm Sephacryl S-500 gel filtration column with a flow rate of 0.1 ml/min (GE Healthcare). The fractions containing the RNPs were collected and affinity purified using amylase resin (27,28), and the purified RNPs were separated by sodium dodecyl sulphate-polyacrylamide gel electrophoresis (SDS-PAGE) and silver stained.

### RNA immunoprecipitations

RNA IP was carried out as previously described (11). ^32^P labeled RNAs were incubated with HeLa nuclear extract for 60 min under standard splicing condition. Five microliters of splicing reaction was mixed with 100 μl of binding buffer (20 mM Hepes at pH 7.9, 200 mM KCl, 0.1% Triton, 2.5 mM ethylenediaminetetraacetic acid [EDTA] and 5 mM Dithiothreitol) and 10 μl of protein A Sepharose beads coupled with 10 μl of antibodies. The mixtures were rotated at 4°C for 2 h. The beads were washed with 1.5 ml of binding buffer for six times. RNAs recovered by phenol/chloroform extraction and ethanol precipitation were analyzed on denaturing polyacrylamide gels and imaged by autoradiography. Twenty-five percent of the input was loaded. Mock depleted, ΔAly, ΔTHO and ΔUAP56/URH49 nuclear extract used for RNA IPs were prepared as previously described (4).

### Protein immunoprecipitations

To carry out IP experiments, 10 μl of antibodies were covalently crosslinked to 20 μl of Protein A beads (crude serum volume: packed beads volume). For IPs from nuclear extract, 75 μl of HeLa nuclear extract was incubated with 50 ng/μl RNase A under splicing condition for 20 min. The reaction mixture was then combined with 125 μl of IP buffer (1× PBS, 0.1% Triton, 0.2 mM phenylmethanesulfonyl fluoride and protease inhibitor [Roche]) and 20 μl of beads crosslinked with antibodies. The mixtures were rotated overnight at 4°C and washed with IP buffer for six times. Proteins were eluted with 30 μl of SDS loading buffer and separated by SDS-PAGE. For IPs from cell lysates, 1 × 10^7^ of HeLa cells transfected with indicated plasmids were harvested and resuspended with 300 μl of binding buffer (20 mM Tris, 100 mM NaCl, 2 mM EDTA, 1 mM DTT, 1 mM PMSF and 0.1% Triton). The sample was sonicated 3 s for four times and centrifuged 12 000 rpm at 4°C for 10 min. The supernatant was incubated with 50 ng/μl of RNase A and protease inhibitor at 30°C for 5 min. After another centrifuge at 12 000 rpm for 10 min at 4°C, the supernatant was added to 20 μl of beads crosslinked with antibodies and rotated overnight at 4°C followed by six washes with binding buffer. Proteins were also eluted with 30 μl of SDS loading buffer and separated by SDS-PAGE.

### Electrophoresis mobility shift assay

^32^P-labeled PRE-SEP1 RNA (12 pmol) or the same amount of size-matched control RNAs were incubated on ice with different molar excesses of purified, bacteria-expressed recombinant GST-ZC3H18-ZF proteins (in 1× PBS, 10% glycerol) for 20 min. Loading buffer (2 μl; 30 mM EDTA, 36% glycerol, 0.05% bromophenol blue, 0.035% xylene cyanol) was added, and the mixtures were separated in a 1% agarose gel (low gelling temperature, Sigma).

### *In vivo* RNA immunoprecipitations

HeLa cells were grown in 10 cm dishes and transfected with indicated siRNAs. Forty-eight hours later, cG-SEP1 plasmid (1 μg/dish), pSUPER-tRNA plasmid (10 μg/dish) and plasmid encoding the VSV M protein (14 μg/dish) were co-transfected into knockdown cells. Twenty-four hours later, cells were harvested and nuclear extracts were prepared as previously described (29). Fifteen microliters of HeLa nuclear extract was incubated under splicing condition for 20 min. 50 μl of reaction mixture was then combined with 25 μl of IP buffer and 20 μl of beads crosslinked with antibodies. IPs were carried out overnight at 4°C. Beads were then washed for six times with the IP buffer. One-fifth of the immunoprecipitates were analyzed by western to determine the IP efficiency of proteins. The rest immunoprecipitates were treated with proteinase K for 1 h at 37°C and the immunoprecipitated RNAs were recovered by phenol/chloroform extraction and ethanol precipitation. The RNAs were then treated with 2U of DNase RQ1 (Promega) for 2 h at 37°C before additional phenol/chloroform extraction and ethanol precipitation. RNAs were reverse transcribed with M-MLV reverse transcriptase (Promega) and cDNA were further quantified by real-time PCR using GoTaq qPCR Master Mix (Promega) according to the manufacturer's protocol.

## RESULTS

### PRE enhances the nuclear export of cDNA transcripts

**Figure 1.**
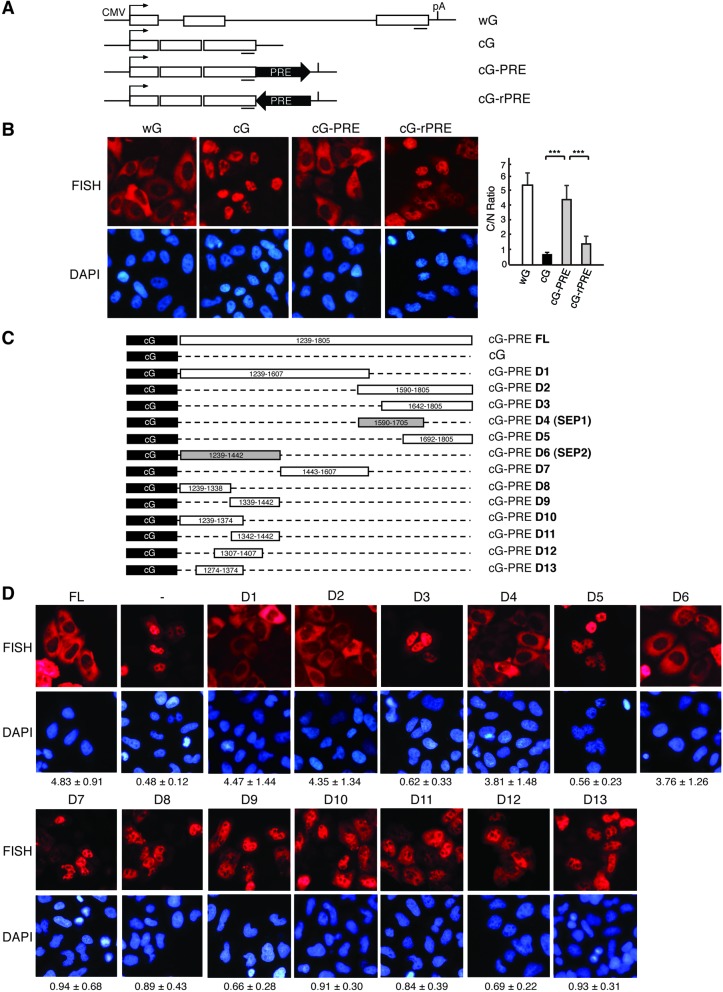
PRE promotes the cytoplasmic accumulation of cDNA transcripts. (**A**) Schematic of the β-globin reporter constructs. The CMV promoter, BGH polyA sites and the location of the FISH probe (vector probe) that detects a region of pcDNA3 vector (indicated by a short line) are shown. (**B**) CMV-DNA constructs were transfected into HeLa cells. Twenty-four hours after transfection, FISH was preformed to detect the indicated mRNAs. DAPI staining was used to indicate the nuclei. C/N ratios were determined for 30 cells per construct in each experiment. The graph shows the average C/N ratios from three independent experiments, and error bars indicate the standard deviations. Statistical analysis was performed using Student's t test. *P < 0.05; **P < 0.01; ***P < 0.001. (**C**) Schematic of the cG constructs with truncated forms of PRE. The remaining parts of PRE are shown with nucleotide numbers relative to the HBV genomic sequence. (**D**) Constructs shown in (C) were transfected into HeLa cells followed by FISH analysis at 24 h after transfection. Numbers below the images represent the C/N ratios (mean value ± SD). Quantification was carried out as in (B).(.

Previous studies suggested that PRE enhances the expression of intronless reporter mRNAs by promoting nuclear export (18). To determine the role of PRE in promoting nuclear export of intronless mRNAs, we constructed β-globin reporter plasmids with PRE inserted in the sense (cG-PRE) or anti-sense direction (cG-rPRE) at the 3′ end of the open reading frame (Figure 1A). These constructs, as well as wild-type β-globin (wG) and its cDNA counterpart (cG), were transfected into HeLa cells, and nucleocytoplasmic distribution of these mRNAs was determined using FISH (Figure 1B). As expected, 24 h after transfection, the wG mRNA mainly accumulated in the cytoplasm, whereas the cG mRNA was mostly retained in the nucleus (Figure 1B, wG and cG). Significantly, the cG-PRE mRNA was largely detected in the cytoplasm. In contrast, the cG-rPRE mRNA was mainly nuclear (Figure 1B). These results indicate that PRE enhances the cytoplasmic accumulation of the cG mRNA. To test whether PRE also enhances cytoplasmic accumulation of other intronless mRNAs, we next used Smad reporter constructs. When PRE was inserted at the 3′ end, the Smad cDNA transcript (cS) that was otherwise retained in the nucleus was accumulated in the cytoplasm to the similar extent to the spliced Smad mRNA (wS) (Supplementary Figure S1). Thus, our data indicate that the role of PRE in enhancing nuclear export of cDNA transcripts is general.

It is well established that splicing promotes mRNA export (3,11,12). To investigate the possibility that PRE enhances mRNA export by inducing a splicing event, the wG, cG and cG-PRE constructs were transfected into HeLa cells, followed by RT-PCRs. As shown in Supplementary Figure S2, for the wG construct, the PCR product that was amplified from the reverse transcript (RT) was approximately 1 kb shorter than that amplified from the plasmid DNA (Supplementary Figure S2). In contrast, similar to what was observed with the cG construct, the sizes of the PCR products for the RT and DNA plasmid of the cG-PRE construct were the same, indicating that no splicing had occurred to the cG-PRE mRNA (Supplementary Figure S2). These results indicate that the enhancement of cytoplasmic accumulation by PRE was not a result of splicing, but rather a direct effect on mRNA export.

### Two export-facilitating sub-elements in PRE

We next sought to examine whether PRE contains multiple export-facilitating sub-elements and to minimize their sizes. To this end, cG plasmids containing differently truncated forms of PRE were constructed and transfected into HeLa cells, followed by FISH analysis. As shown in Figure 1C, when PRE was separated into two fragments that only have 18 nt overlapping sequence, both efficiently promoted the nuclear export of the cG mRNA (Figure 1C and D, D1 and D2). We subsequently shortened these two fragments. The D2 fragment was successfully shortened to 116 nt (SEP1) without significantly reducing its export activity (Figure 1D, D4). In contrast, we could only shorten the D1 fragment to 204 nt (SEP2; Figure 1D, D6-D13). Nevertheless, our results indicate that PRE contains at least two independent, export-facilitating sub-elements. This is consistent with a previous study which reported that PRE contains two sub-elements (263 and 333 nt, respectively) that function synergistically in facilitating the expression of cDNA transcripts (30). Considering that shorter RNA elements usually have better specificities for the identification of associating proteins, we used SEP1 for further studies.

### Purification of the RNP formed on the SEP1 RNA

**Figure 2.**
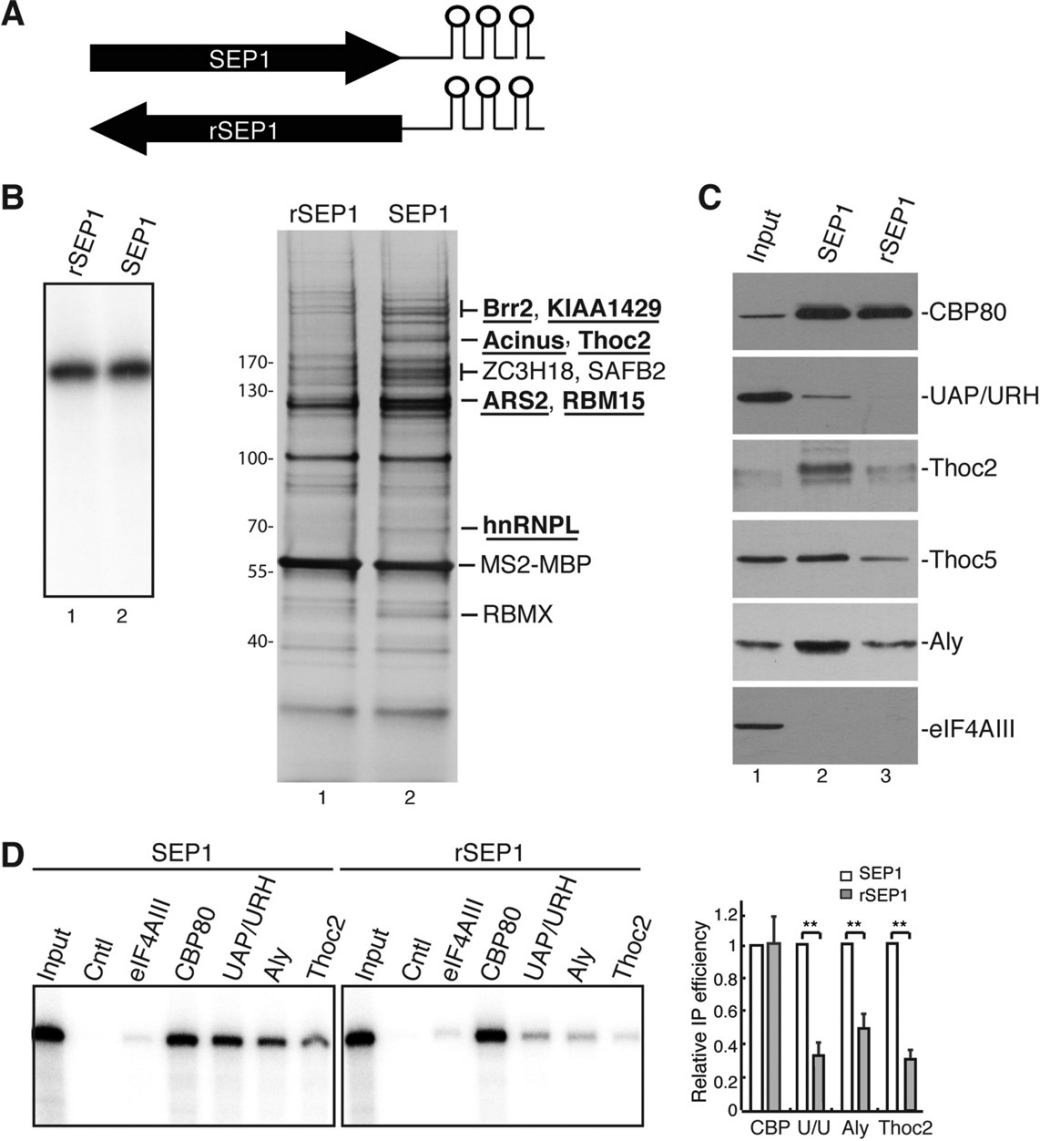
Purification and identification of SEP1-interacting proteins. (**A**) Schematic of the RNA substrates that were used for RNP purifications. Each stem loop indicates an MS2 binding site. (**B** and **C**) *In vitro* transcribed, ^32^P-labeled SEP1 and its reverse complement sequence (rSEP1) containing three MS2 binding sites were incubated with MS2-MBP followed by incubation with HeLa nuclear extract for 1 h. The mixtures were separated by gel filtration and purified using amylose resin. The purified RNPs were then separated by SDS-PAGE followed by silver-staining (B) and western analyses (C) using the indicated antibodies. RNAs that were present in the purified RNPs were visualized using autoradiography and are shown in the left panel in (B). The proteins present in the bands that are indicated in the right panel in (B) were identified using mass spectrometry. The UAP/URH antibody detects both UAP56 and URH49. (**D**) *In vitro* transcribed, ^32^P-labeled SEP1 and rSEP1 were incubated in HeLa nuclear extract under splicing conditions for 1 h followed by RNA IPs using the indicated antibodies. The control was an antibody to fSAP130. One-fourth of the input was loaded. The graph shows the quantification of IP efficiencies. U/U indicates UAP56/URH49. The bars indicate the average ratios of IP efficiencies for rSEP1 relative to those for SEP1 for three independent experiments.

To identify the cellular proteins that specifically associate with the SEP1 RNA, SEP1 and its reverse complement sequence (rSEP1) were fused to three MS2 binding sites and *in vitro* transcribed (Figure 2A). These RNAs were incubated with MS2-MBP, followed by incubation with HeLa nuclear extract under splicing conditions for 60 min. RNPs formed on the SEP1 and rSEP1 RNAs were isolated by gel filtration followed by affinity purification using amylose resin. Proteins from equivalent amounts of RNPs were separated by SDS-PAGE followed by silver staining. As shown in Figure 2B, multiple proteins were specifically present in the SEP1 RNP. These proteins were identified using mass spectrometry. Strikingly, most of these proteins were TREX components or putative TREX-interacting proteins that include Brr2, KIAA1429, Acinus, ARS2, RBM15 and hnRNPL (Figure 2B, labeled underline) (3,9,31). In addition, three putative RNA-binding proteins SAFB2, ZC3H18 and RBMX were also specifically detected in the SEP1 RNP. To examine whether the entire TREX complex associates with the SEP1 RNA, western analyses were carried out on SEP1 and rSEP1 RNPs using antibodies to TREX proteins. CBP80 was used as a loading control, and eIF4AIII, which is a component of the EJC, was used to determine the specificity of the purifications. As shown in Figure 2C, CBP80 was present equally in the SEP1 and rSEP1 RNP, whereas eIF4AIII was absent from both RNPs. Significantly, UAP56/URH49 was readily detected in the SEP1 RNP, but not in the rSEP1 RNP (Figure 2C). The amounts of Thoc2, Thoc5 and Aly present in the SEP1 RNP were also significantly more than those in the rSEP1 RNP. These results indicate that the entire TREX complex efficiently associated with the SEP1 RNA.

To further investigate the association of TREX with the SEP1 RNA, we next carried out RNA immunoprecipitations (IPs). *In vitro* transcribed SEP1 and rSEP1 RNAs were incubated in HeLa nuclear extract under splicing conditions followed by IPs with antibodies to CBP80, Aly, UAP56/URH49 and Thoc2 (Figure 2D). eIF4AIII and fSAP130 antibodies were used as negative controls. As shown in Figure 2D, the CBP80 antibody equally immunoprecipitated (IP’d) the SEP1 and rSEP1 RNAs, whereas the negative control antibodies did not immunoprecipitate either RNA. Significantly, the SEP1 RNA, but not the rSEP1 RNA, was efficiently IP'd by the Aly, UAP56/URH49 and Thoc2 antibodies (Figure 2D), indicating that the SEP1 RNA is specifically bound by TREX proteins. Taken together, we conclude that TREX proteins specifically associate with the SEP1 RNA.

### TREX is required for PRE-mediated mRNA export

**Figure 3.**
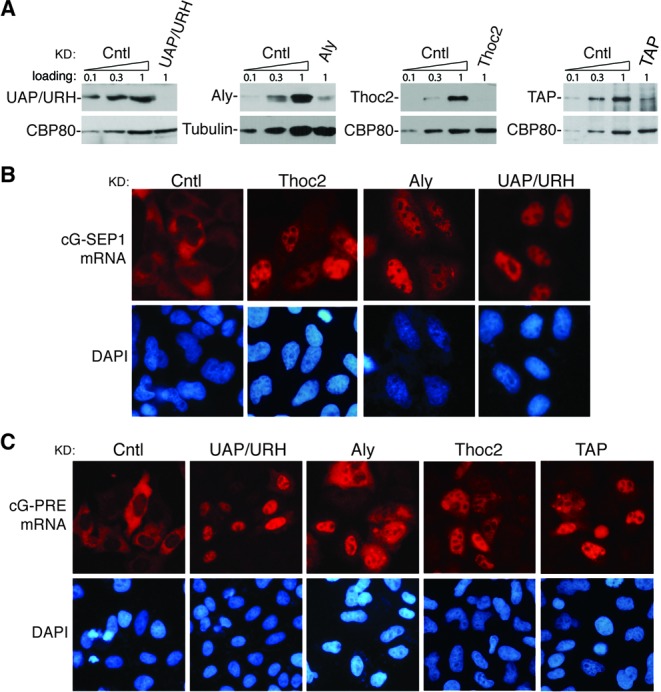
TREX proteins and TAP play critical roles in PRE-mediated mRNA export. (**A**) HeLa cells were transfected with siRNAs targeting the indicated genes, and lysates were prepared 60 h after transfection. Western analyses of the cell lysates were carried out with the indicated antibodies. Control knockdown cell lysates (10%, 30% and 100%) were loaded. (**B**) The cG-SEP1 construct was transfected into HeLa cells 36 h after siRNA transfection, and 24 h later, FISH was carried out to detect the cG-SEP1 mRNA. The FISH probe was same as that used in Figure 1. (**C**) Similar to (B), except that the cG-PRE construct was transfected, and TAP knockdown cells were also included.

The fact that TREX specifically associates with the SEP1 RNA raised the possibility that it plays a role in SEP1-dependent mRNA export. To test this possibility, we carried out RNA­-mediated interference (RNAi) of UAP56/URH49, Aly and Thoc2 in HeLa cells and used a non­targeting siRNA as a negative control. Western analyses revealed that protein levels of these genes were efficiently knocked down (Figure 3A). When the cG-SEP1 construct was transfected into control knockdown cells, the corresponding mRNA was mainly detected in the cytoplasm. In marked contrast, the cG-SEP1 mRNA was mostly retained in the nuclei in UAP56/URH49-, Aly- and Thoc2-knockdown cells (Figure 3B). These results indicate that TREX plays key roles in SEP1-mediated mRNA export.

It is possible that other sub-elements in PRE, such as SEP2, recruit other RNA export adaptors/receptors that play redundant roles with TREX in PRE-mediated mRNA export. If this were true, one would expect that TREX is not necessary for nuclear export mediated by the full-length PRE. To test this possibility, we examined the role of TREX and the mRNA export receptor TAP in PRE-mediated mRNA export. As shown in Figure 3C, although the cG-PRE mRNA was mostly cytoplasmic in control knockdown cells, it was largely retained in the nuclei of Aly-, Thoc2-, UAP56/URH49- and TAP-knockdown cells (Figure 3C). Together, these results indicate that TREX is required for PRE-mediated mRNA export and PRE utilizes the cellular mRNA export pathway.

### ZC3H18 plays key roles in SEP1-dependent mRNA export

**Figure 4.**
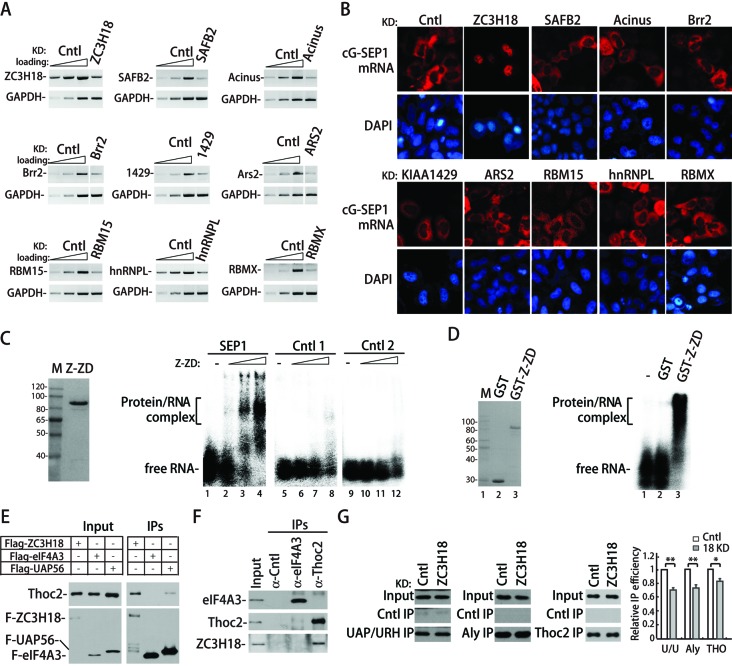
ZC3H18 plays key roles in SEP1-dependent mRNA export. (**A**) HeLa cells were infected with lenti-viruses expressing the indicated shRNAs, and 96 h later, total RNAs were extracted followed by RT-PCR analyses to determine knockdown efficiencies. RT products of the control knockdown (10%, 30% and 100%) were used for PCR. (**B**) Cells described in (A) were transfected with the cG-SEP1 construct at 72 h post-infection, followed by FISH analysis 24 h later to detect the cG-SEP1 mRNA. The FISH probe was same as that used in Figure 1. (**C**) 12 pmol of ^32^P-labeled SEP1 and two control RNAs were incubated with 0, 12, 40 and 120 nmol of purified GST-ZC3H18-ZD (Z-ZD). The protein–RNA complexes were fractionated on a 1% agarose gel and visualized using autoradiography. The position of free RNAs and protein-bound RNAs are indicated. The left panel shows coomassie staining of the purified Z-ZD protein. (**D**) ^32^P-labeled SEP1 (12 pmol) were incubated with 200 nmol of purified GST or GST-Z-ZD protein followed by electrophoresis mobility shift assay similar to (C). The left panel shows coomassie staining of the purified GST and GST-Z-ZD protein. (**E**) Plasmids encoding the indicated Flag-tagged proteins were transfected into HeLa cells, and 24 h later, the cells were harvested and RNase A-treated lysates were used for Flag-IPs followed by western analyses using Thoc2 and Flag antibodies. (**F**) The pcDNA3-ZC3H18 plasmid was transfected into HeLa cells, and 24 h later, the cells were harvested and RNase A-treated lysates were used for IPs using Thoc2 and eIF4A3 antibodies followed by western analyses with indicated antibodies. (**G**) HeLa cells were treated with control or ZC3H18 siRNA. Forty-eight hours later, cG-SEP1, pSUPER-tRNA and VSV M plasmids were co-transfected into knockdown cells. Twenty-four hours later, cells were harvested and *in vivo* RNA immunoprecipitation was carried out using indicated antibodies. Immunoprecipitated RNAs were analyzed by RT-qPCR. The bars indicate the ratio of the IP efficiency of cG-SEP1 mRNA relative to that of tRNA. The relative IP efficiencies from control knockdown cells were considered as 100%. The bars and error bars represent average values and standard deviations from three independent experiments. Immunoprecipitated proteins were analyzed by westerns (left panel).

We next examined the roles of other SEP1-associating proteins in SEP1-mediated mRNA export. To this end, we carried out RNAi of these proteins, including Brr2, KIAA1429, Acinus, ARS2, hnRNPL, RBM15, RBMX, SAFB2 and ZC3H18. RT-PCR analyses showed that mRNA levels of the target genes were reduced at least 70% (Figure 4A). When RBMX, SAFB2 as well as previously reported TREX-interacting proteins were knocked down, the nucleocytoplasmic distribution of the cG-SEP1 mRNA was not significantly affected (Figure 4B). However, unexpectedly, knockdown of ZC3H18, which was not known to interact with TREX, significantly inhibited nuclear export of the cG-SEP1 mRNA. To confirm this result, we used three different siRNAs that efficiently silenced the expression of ZC3H18 (Supplementary Figure S3). Similar to shRNA-treated cells, the cG-SEP1 mRNA was largely nuclear in ZC3H18 siRNA-treated cells. Therefore, we concluded that ZC3H18 is required for SEP1-mediated mRNA export.

We also examined the role of ZC3H18 in mRNA export mediated by the full-length PRE. As shown in Supplementary Figure S3, knockdown of ZC3H18 did not significantly affect nuclear export of the cG-PRE mRNA, suggesting that other sub-elements in PRE, like SEP2, promote nuclear export of intronless mRNAs via a mechanism independent of ZC3H18.

### ZC3H18 directly binds the SEP1 RNA, interacts with TREX and is required for efficient TREX recruitment to the SEP1 RNA

ZC3H18 contains a CCCH zinc finger domain that has the potential for RNA-binding. We next examined whether ZC3H18 directly binds to the SEP1 RNA. We expressed the recombinant GST-tagged zinc finger domain of ZC3H18 (GST-Z-ZD; Figure 4C, left panel) in bacteria and employed electrophoresis mobility shift assays. A size matched RNA (Cntl 1) that can also enhance nuclear export of intronless mRNAs was used as a control (Supplementary Figure S4). In addition, the anti-sense transcript of Cntl 1 RNA (Cntl 2) was also included. ^32^P-labeled SEP1 and control RNAs were incubated with increasing amounts of GST-Z-ZD. More than 50% of binding to the SEP1 RNA was observed at 40 nM of Z-ZD (Figure 4C, lane 3), whereas no significant RNA–protein complex formation with the control RNAs was detectable (Figure 4C). To further examine the specificity of the interaction between ZC3H18 and the SEP1 RNA, we next incubated the SEP1 RNA with equal amount of GST or GST-Z-ZD. As shown in Figure 4D, the SEP1 RNA form RNA–protein complexes with GST-Z-ZD, but not with GST itself. These data show that recombinant Z-ZD binds directly to the SEP1 RNA, which is in agreement with our RNP purification results and demonstrated that ZC3H18 preferentially binds the SEP1 RNA.

It is possible that ZC3H18 binds to the SEP1 RNA directly and recruits TREX. If this were true, one would expect that ZC3H18 interacts with TREX. To test this interaction, we examined whether exogenously expressed Flag-ZC3H18 co-immunoprecipitates TREX components. Flag-eIF4AIII and Flag-UAP56 were used as negative and positive controls, respectively. As expected, the TREX protein Thoc2 was co-immunoprecipitated by Flag-UAP56, but not by Flag-eIF4AIII (Figure 4E). As shown in Figure 4E, although the level of Flag-ZC3H18 was apparently lower than that of Flag-UAP56, significantly more Thoc2 was co-IP'd with Flag-ZC3H18 than Flag-UAP56. This result indicates that ZC3H18 strongly associates with Thoc2. To confirm this association, we carried out reverse IPs from the cell lysates expressing exogenous ZC3H18 using a Thoc2 antibody and a control antibody to eIF4AIII. As shown in Figure 4F, ZC3H18 was present in the immunoprecipitate of the Thoc2 antibody, but not in that of the eIF4AIII antibody. These results indicate that ZC3H18 associates with the TREX component Thoc2.

We next asked whether ZC3H18 functions in recruiting TREX to the SEP1-containing mRNA. To answer this question, HeLa cells were treated with ZC3H18 or the control siRNA followed by co-transfection of the cG-SEP1 construct with a control tRNA construct. IPs were carried out with antibodies to Aly, UAP56/URH49 and Thoc2. RNAs coprecipitated were extracted, reverse transcribed and amplified by qPCRs. As shown in Figure 4G, moderate but reproducible decrease in the levels of cG-SEP1 mRNA associating with TREX proteins were observed with ZC3H18 knockdown compared to the control knockdown. This decrease was not a result of different TREX IP efficiencies in control and ZC3H18 knockdown, as equal amount of TREX proteins were present in immunoprecipitates from these two sets of knockdown cells (Figure 4G). This result indicates that ZC3H18 functions in TREX recruitment to SEP1-containing mRNAs. Together, our data indicate that ZC3H18 specifically binds to the SEP1 RNA and recruits TREX to SEP1-contiaing mRNAs via protein–protein interactions.

### Efficient TREX recruitment to the SEP1 RNA depends on the 5′ cap and TREX integrity

**Figure 5.**
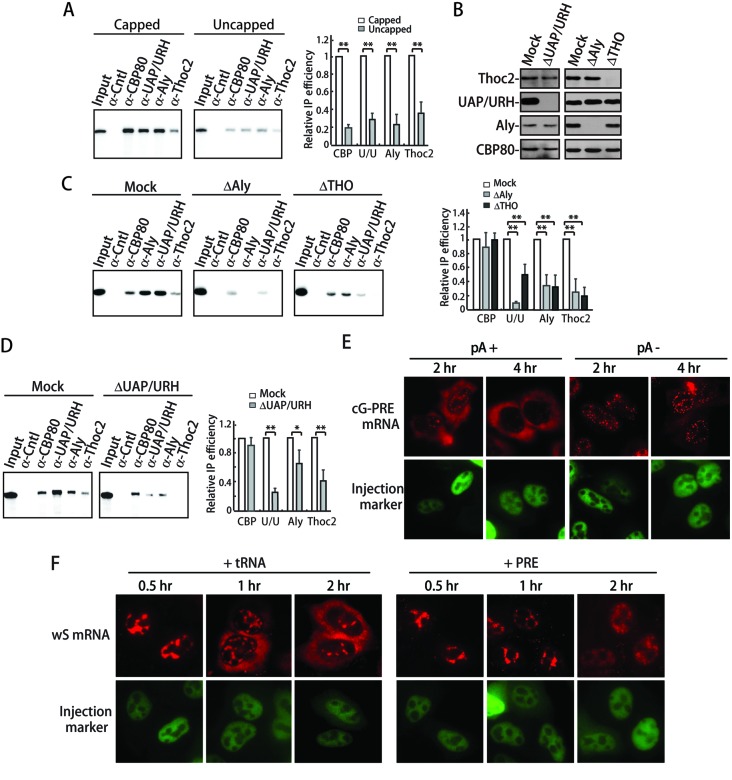
Requirements for SEP1-mediated RNA export are highly similar to those for cellular mRNA export. (**A**) *In vitro* transcribed, ^32^P-labeled capped and uncapped SEP1 were incubated in HeLa nuclear extract under splicing condition for 1 h followed by RNA IPs with the indicated antibodies. The right graph shows quantification of IP efficiencies from three independent experiments. The bars indicate the ratios of IP efficiencies using uncapped SEP1 RNAs relative to those with capped SEP1 RNAs. (**B**) Western analyses of immunodepleted HeLa nuclear extracts using the indicated antibodies. (**C**) *In vitro* transcribed, ^32^P-labeled SEP1 was incubated in mock-, Aly- or Thoc2-immunodepleted nuclear extracts (Mock, ΔAly and ΔTHO extracts) under splicing conditions for 1 h followed by RNA IPs using the indicated antibodies. The right graph shows quantification of three independent experiments. The bars indicate the ratio of the IP efficiencies of ΔAly or ΔTHO extracts relative to the corresponding IP efficiency in the mock extract. (**D**) Same as (C), except that the ΔUAP56/URH49 extract was used. (**E**) The fragment from the CMV promoter to the polyadenylation signal of the cG-PRE plasmid with a WT (pA+) or mutated polyadenylation signal (pA-) was amplified by PCR. PCR products (200 ng/μl) were microinjected into HeLa nuclei, and α-amanitin was added to block transcription 15 min after microinjection. FISH was carried out at the indicated time point to detect the distribution of the cG-PRE mRNAs. The FISH probe was same as that used in Figure 1. (**F**) The wS construct (50 ng/μl) was co-injected with 200 ng/μl of PRE plasmid or tRNA plasmid into HeLa nuclei, and α-amanitin was added to block transcription 15 min after microinjection. FISH was carried out to detect the distribution of Smad mRNAs at 0.5, 1 and 2 h after injection using Smad probe.

The moderate effect of ZC3H18 knockdown on TREX recruitment to the SEP1 RNA suggests that ZC3H18 might not be the only factor recruiting TREX. Previously we had showed that splicing-dependent TREX recruitment requires the 5′ cap (4,11). We next examined whether the 5′ cap/CBC plays a role in TREX recruitment to the SEP1 RNA. To do this, we *in vitro* synthesized capped and uncapped SEP1 RNA followed by RNA IPs with antibodies to CBP80, Aly, UAP56/URH49 and Thoc2. As expected, capped, but not uncapped, SEP1 RNA was efficiently IP'd by the CBP80 antibody. Significantly, Aly, UAP56/URH49 and Thoc2 efficiently associated with the capped, but not the uncapped SEP1 RNA (Figure 5A). This result indicates that ZC3H18 is not the only requirement for SEP1-dependent TREX recruitment and the 5′ cap/CBC is also required for SEP1-mediated TREX recruitment.

Aly and THO are required for the efficient recruitment of TREX proteins to spliced mRNAs (4). However, the role of UAP56/URH49 in TREX recruitment remains to be determined. To examine whether Aly, UAP56/URH49 and THO is required for recruitment of one another to the SEP1 RNA, we carried out RNA IPs from Aly-, THO- and UAP56/URH49-immunodepleted HeLa nuclear extracts (ΔAly, ΔTHO and ΔUAP56/URH49 extracts, respectively). The mock-depleted nuclear extract (Mock extract) was used as a control. Aly, THO and UAP56/URH49 were efficiently depleted without significantly affecting the levels of the other proteins (Figure 5B). In the Mock extract, the SEP1 RNA was efficiently IP'd by antibodies to CBP80, Aly, UAP56/URH49 and Thoc2 (Figure 5C and D). In contrast, depletion of Aly or THO inhibited the association of other TREX proteins with the SEP1 RNA (Figure 5C and D). Although UAP56/URH49 could not be completely depleted, as judged from the small amount of SEP1 RNA that was IP'd by the UAP56/URH49 antibody in the ΔUAP56/URH49 extract, a moderate but reproducible reduction of the SEP1 RNA associated with Aly and Thoc2 was observed (Figure 5D). These data show that Aly, THO and UAP56/URH49 require one another to efficiently associate with the SEP1 RNA. Together, we conclude that requirements for TREX recruitment to the SEP1 RNA are highly similar to those for TREX recruitment to spliced mRNAs.

### 3′ end processing is required for PRE-mediated mRNA export

3′ end processing is required for the efficient export of spliced mRNAs (32). To test whether PRE-mediated mRNA export also requires appropriate 3′ end processing, the AATAAA in the polyA signal of the cG-PRE construct was mutated to GGATCC, and the construct was microinjected into nuclei of HeLa cells followed by FISH. As shown in Figure 5E, the cG-PRE mRNA with the WT polyA signal partially accumulated in the cytoplasm 2 h post-injection and was mostly cytoplasmic 4 h later. In marked contrast, the cG-PRE mRNA with mutated polyA signal was mainly nuclear even 4 h after microinjection. These results indicate that similar to cellular spliced mRNAs, 3′ end processing is also required for PRE-dependent mRNA export.

### PRE competes with spliced mRNAs for nuclear export

The fact that the nuclear export of PRE-containing RNAs share common features with that of spliced mRNAs suggest that these two pathways may compete with each other. To test this possibility, we next asked whether PRE has any *trans* effect on the nuclear export of spliced mRNAs. To answer this question, the wS plasmid was co-injected with excess amounts of plasmid expressing PRE or tRNA into the nuclei of HeLa cells, and the effect on the distribution of the wS mRNA was examined. When the tRNA construct was co-injected, the wS mRNA began to accumulate in the cytoplasm at 0.5 h, and the cytoplasmic wS mRNA gradually increased at the 1 h and 2 h time points (Figure 5F, +tRNA). In contrast, the wS mRNA was only detected in the nuclei of cells co-injected with PRE, even at 2 h post-injection (Figure 5F, +PRE). This result indicates that PRE indeed competes with spliced mRNAs for nuclear export.

### ZC3H18, ARS2, Acinus and Brr2 are required for cellular mRNA export

**Figure 6.**
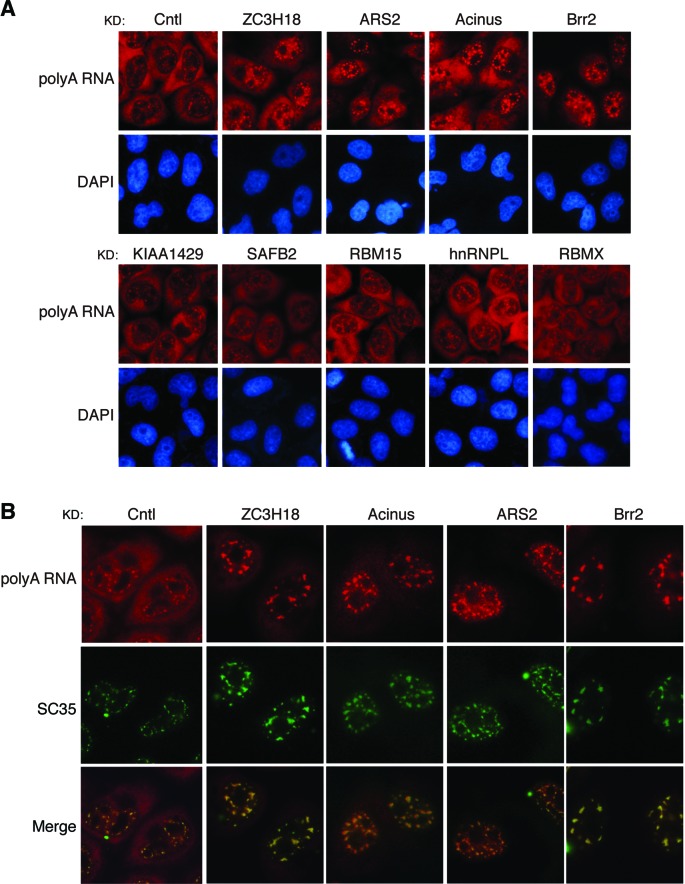
ZC3H18, Ars2, Acinus and Brr2 are required for cellular mRNA export. (**A**) HeLa cells were infected with lenti-viruses expressing the indicated shRNAs. Ninety-six hours later, FISH was carried out to determine the distribution of the polyA RNAs using an oligo-dT (70) probe. (**B**) HeLa cells were transfected with the indicated siRNAs. Seventy-two hours later, polyA FISH and SC35 immunofluorescence (IF) were carried out to detect the localization of polyA RNAs and SC35. The polyA FISH, SC35 IF and merged images are shown.

The competition between PRE and spliced mRNA prompted us to test whether SEP1-associating proteins play any role in the nuclear export of cellular mRNAs. To this end, we examined how the nucleocytoplasmic distribution of polyA RNAs is affected by knockdown of these proteins. In control shRNA-treated cells, polyA RNAs were mainly detected in the cytoplasm. Similarly, in SAFB2-, KIAA1429-, hnRNPL-, RBM15- and RBMX-knockdown cells, polyA RNAs were also mainly accumulated in the cytoplasm. In contrast, in ZC3H18-, Acinus-, ARS2- and Brr2-knockdown cells, significant amount of polyA RNAs was detected in the nucleus (Figure 6A). To confirm these results, we used three different siRNAs to knock down each of these genes. RT-PCR/western analyses showed that for ZC3H18, Acinus and Brr2, all three siRNAs knocked down the target genes efficiently, whereas for ARS2, only two siRNAs efficiently reduced its expression level (Supplementary Figures S3 and S5). Correlated with the knockdown efficiencies, polyA RNAs were detected at least partially, if not mostly, in the nuclei of these siRNA-treated cells, except for cells treated with the ARS2-3 siRNA, which did not efficiently knock down ARS2 (Supplementary Figure S5). Together, these results indicate that ZC3H18, ARS2, Acinus and Brr2 are required for efficient nuclear export of cellular mRNAs.

It is known that the entire TREX complex is required to release mRNAs from the nuclear speckle domains that are enriched with splicing factors (4,26). Previously, ARS2, Acinus and Brr2 were found in the spliceosome and pulled down by and/or IP'd with TREX proteins (3,9,33,34). Thus, it is possible that these proteins may function in cellular mRNA export by recruiting TREX during splicing. If this were true, silencing the expression of these proteins would also lead to the accumulation of polyA RNAs in nuclear speckle domains. To investigate this possibility, we carried out a co-localization study of nuclear-accumulated polyA RNAs with SC35, which is the standard marker for nuclear speckle domains (Figure 6B) (26). In control knockdown cells, as expected, a small portion of polyA RNAs were present in nuclear speckle domains. In contrast, in ARS2-, Acinus- and Brr2-knockdown cells, significant amount of polyA RNAs was detected in the nucleus and these nuclear-accumulated polyA RNAs indeed generally co-localized with SC35. Interestingly, in ZC3H18-knockdown cells, polyA RNAs were also retained in nuclear speckle domains. These data are consistent with the possibility that ARS2, Acinus, Brr2 as well as ZC3H18 are involved in TREX recruitment during splicing.

## DISCUSSION

In higher eukaryotes, the TREX complex, which functions in mRNA export, is recruited to spliced mRNAs dependent upon splicing and to naturally intronless mRNAs dependent upon *cis*-acting RNA elements that are present in these mRNAs. However, the mechanism for neither splicing-dependent nor element-dependent recruitment of TREX is well understood. In this study, we found that a functional sub-element of PRE (SEP1) can replace the role of splicing in TREX recruitment. Our data support a model where SEP1 is recognized by ZC3H18, which subsequently recruits TREX to SEP1-containing intronless mRNAs. In addition, we found that several cellular proteins that associate with PRE are required for efficient cellular mRNA export. According to their reported roles and interacting partners, these proteins might be involved in cap- and splicing-dependent TREX recruitment to cellular mRNAs.

Several lines of evidence support the model for ZC3H18-dependent TREX recruitment to SEP1-containing mRNAs. First, ZC3H18 and TREX proteins were identified in the RNP that is formed on the SEP1 RNA. Second, ZC3H18 and TREX are required for SEP1-mediated mRNA export. Third, ZC3H18 directly binds the SEP1 RNA and interacts with TREX via protein–protein interactions. Finally, knockdown of ZC3H18 results in reduced association of TREX proteins with the SEP1-containing mRNA. However, ZC3H18 is not the only requirement for stable association of TREX with the SEP1 RNA. Both the 5′ cap and 3′ end processing are required for SEP1-dependent TREX recruitment or mRNA export. Although most intronless mRNAs do not undergo splicing, they do need to be capped and polyadenylated prior to nuclear export. Thus, the possibility that ZC3H18, the 5′ cap/CBC and the 3′ end processing machineries all contribute to TREX recruitment to SEP1-containing mRNAs would ensure that only fully processed mRNAs can be exported. In contrast to the moderate effect of ZC3H18 knockdown on TREX recruitment, we did observe a significant inhibition on SEP1-dependent mRNA export. It is possible that moderate reduction of TREX recruitment is enough to cause significant inhibition on mRNA export.

Previously, it was concluded that PRE does not use the mRNA export pathway by demonstrating that a mutant RanBP1 inhibits CAT expression dependent on PRE, but not that dependent on CTE (19). However, the decreased expression can also be explained by reduced transcription or translation, or elevated mRNA degradation. Consistent with possible enhanced mRNA degradation, the CAT mRNA levels in both the nucleus and the cytoplasm decreased significantly when the mutant RanBP1 was expressed (19). Here, we found that knockdown of TREX or TAP blocked PRE-dependent mRNA export and excess amount of PRE inhibited mRNA export, demonstrating that PRE utilizes the mRNA export pathway. Notably, the mechanism for SEP1 enhancing mRNA export is distinct from other known viral RNA elements that utilize TREX for nuclear export. Kaposi's Sarcoma-associated herpesvirus (KSAH), herpes simplex virus type 1 (HSV) and the human cytomegalovirus all encode specific viral proteins that bind viral mRNAs and recruit TREX to viral unspliced mRNAs (35–38). In contrast, the role of SEP1 in mRNA export does not require any viral protein, but is dependent upon the cellular protein ZC3H18. Thus, although SEP1 is a viral RNA element, the mechanism by which it enhances mRNA export is rather similar to that for *cis*-acting RNA elements promoting nuclear export of naturally intronless mRNAs.

To date, our understanding of the mechanisms by which *cis*-acting RNA elements recruit TREX to export cellular intronless mRNAs is very limited. The Prp19 complex and U2AF2 have been shown to associate with some intronless mRNAs and function in their export (39). However, it remains unknown whether these proteins directly bind to these mRNAs. It is possible that some common mRNA export factors function in nuclear export mediated by different *cis*-acting RNA elements. Consistent with this possibility, ZC3H18 was also found to preferentially associate with RNA elements that were identified in some intronless mRNAs (39). Therefore, it is possible that ZC3H18 functions in the nuclear export of naturally intronless mRNAs that contain SEP1-like RNA elements. Our result showing that knockdown of ZC3H18 led to partial nuclear retention of polyA RNAs suggests that ZC3H18 is required for the export of a subset of cellular mRNAs. It would be interesting to investigate whether the nuclear retained polyA RNAs include naturally intronless mRNAs that contain SEP1-like elements.

Two major possible mechanisms have been proposed for splicing-dependent TREX recruitment. One possibility is that some splicing factors that interact with TREX proteins function in their recruitment during splicing. Our result demonstrating that the U5 snRNP component Brr2 functions in mRNA export supports this possibility. In a previous study, we found that different from Aly and THO, which exclusively associate with spliced mRNAs, UAP56/URH49 also associates with pre-mRNAs via an unknown mechanism (4). Interestingly, Brr2 was specifically enriched in UAP56/URH49 immunoprecipitate, but not in those of other TREX proteins (9). Thus, it is possible that during splicing, Brr2 recruits UAP56/URH49 to pre-mRNAs via protein–protein interaction. Some other splicing factors might also be involved in recruiting UAP56/URH49 to pre-mRNAs. For example, the Prp19 complex also interacts with UAP56 and functions in mRNA export in yeast (9,40). Another possible mechanism for splicing-dependent TREX recruitment is that components of the spliced mRNP, such as the EJC, stabilize TREX on spliced mRNAs. In agreement with this possibility, we found that the EJC component Acinus, which was previously pulled down by Aly, is required for the efficient export of polyA RNAs (3,41). Acinus might possibly function in mRNA export by stabilizing TREX on spliced mRNAs. Obviously, these two mechanisms are not mutually exclusive, but might function synergistically, which could explain why knockdown of Brr2 and Acinus alone only led to moderate mRNA export blockage. Further studies on the roles of Brr2 and Acinus in TREX recruitment to spliced mRNA are needed. Except for Brr2 and Acinus, other factors and mechanisms might also be involved in splicing-dependent mRNA export.

**Figure 7.**
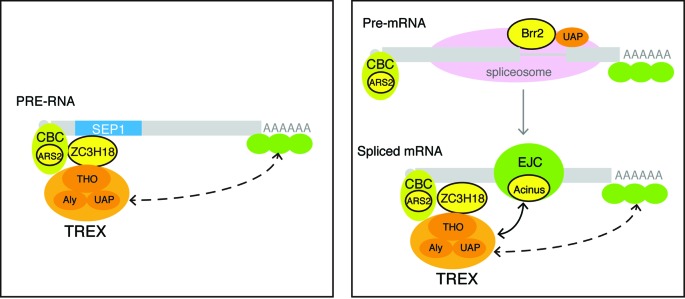
Models for the roles of SEP1-associating proteins in the nuclear export of PRE-containing mRNAs and spliced cellular mRNAs. Left panel: ZC3H18 directly binds to the SEP1 RNA, and TREX is recruited to the SEP1 RNA via ZC3H18. Right panel: possible roles of ARS2, ZC3H18, Acinus and Brr2 in TREX recruitment to cellular mRNAs during splicing (see the details in the Discussion section). In both situations, CBC and 3′ end processing factors are required for recruiting/stabilizing TREX to the mRNAs to form export-competent RNPs.

Previously, it has been shown that in addition to splicing, TREX recruitment is dependent upon the 5′ cap (11). It is thought that this recruitment occurs via interactions between the cap-binding protein CBP80 and the TREX proteins Aly and Thoc2 (4). However, knockdown of CBP80 and CBP20 does not have an apparent effect on mRNA export (Chi,B., unpublished observations). ARS2 is a recently identified component of the nuclear CBC that was enriched in TREX IPs (3,9,42) and has been implicated in 3′ end processing and degradation of mRNAs (43,44). Considering the significant inhibitory effect of ARS2 knockdown on mRNA export, it is possible that it plays a more important role than CBP80 in recruiting TREX (Figure 7, right panel). A recent study reported that ZC3H18 interacts with CBP80/CBP20 and ARS2 (43). Thus, it is possible that ZC3H18 together with CBC recruits TREX to spliced mRNAs and thus functions in cap-dependent TREX recruitment and mRNA export. Further studies on the interactions of CBP80, ARS2, ZC3H18 and TREX might refine the current model for cap-dependent TREX recruitment.

## SUPPLEMENTARY DATA

Supplementary Data are available at NAR Online.

SUPPLEMENTARY DATA

## References

[1] Katahira J., Inoue H., Hurt E., Yoneda Y. (2009). Adaptor Aly and co-adaptor Thoc5 function in the Tap-p15-mediated nuclear export of HSP70 mRNA. EMBO J..

[2] Viphakone N., Hautbergue G.M., Walsh M., Chang C.T., Holland A., Folco E.G., Reed R., Wilson S.A. (2012). TREX exposes the RNA-binding domain of Nxf1 to enable mRNA export. Nat. Commun..

[3] Masuda S., Das R., Cheng H., Hurt E., Dorman N., Reed R. (2005). Recruitment of the human TREX complex to mRNA during splicing. Genes Dev..

[4] Chi B., Wang Q., Wu G., Tan M., Wang L., Shi M., Chang X., Cheng H. (2013). Aly and THO are required for assembly of the human TREX complex and association of TREX components with the spliced mRNA. Nucleic Acids Res..

[5] Jimeno S., Rondon A.G., Luna R., Aguilera A. (2002). The yeast THO complex and mRNA export factors link RNA metabolism with transcription and genome instability. EMBO J..

[6] Strasser K., Masuda S., Mason P., Pfannstiel J., Oppizzi M., Rodriguez-Navarro S., Rondon A.G., Aguilera A., Struhl K., Reed R. (2002). TREX is a conserved complex coupling transcription with messenger RNA export. Nature.

[7] Rehwinkel J., Herold A., Gari K., Kocher T., Rode M., Ciccarelli F.L., Wilm M., Izaurralde E. (2004). Genome-wide analysis of mRNAs regulated by the THO complex in Drosophila melanogaster. Nat. Struct. Mol. Biol..

[8] Yamazaki T., Fujiwara N., Yukinaga H., Ebisuya M., Shiki T., Kurihara T., Kioka N., Kambe T., Nagao M., Nishida E. (2010). The closely related RNA helicases, UAP56 and URH49, preferentially form distinct mRNA export machineries and coordinately regulate mitotic progression. Mol. Biol. Cell.

[9] Dufu K., Livingstone M.J., Seebacher J., Gygi S.P., Wilson S.A., Reed R. (2010). ATP is required for interactions between UAP56 and two conserved mRNA export proteins, Aly and CIP29, to assemble the TREX complex. Genes Dev..

[10] Chang C.T., Hautbergue G.M., Walsh M.J., Viphakone N., van Dijk T.B., Philipsen S., Wilson S.A. (2013). Chtop is a component of the dynamic TREX mRNA export complex. EMBO J..

[11] Cheng H., Dufu K., Lee C.S., Hsu J.L., Dias A., Reed R. (2006). Human mRNA export machinery recruited to the 5’ end of mRNA. Cell.

[12] Valencia P., Dias A.P., Reed R. (2008). Splicing promotes rapid and efficient mRNA export in mammalian cells. Proc. Natl. Acad. Sci. U.S.A..

[13] Neville M., Stutz F., Lee L., Davis L.I., Rosbash M. (1997). The importin-beta family member Crm1p bridges the interaction between Rev and the nuclear pore complex during nuclear export. Curr. Biol..

[14] Fornerod M., Ohno M., Yoshida M., Mattaj I.W. (1997). CRM1 is an export receptor for leucine-rich nuclear export signals. Cell.

[15] Gruter P., Tabernero C., von Kobbe C., Schmitt C., Saavedra C., Bachi A., Wilm M., Felber B.K., Izaurralde E. (1998). TAP, the human homolog of Mex67p, mediates CTE-dependent RNA export from the nucleus. Mol. Cell.

[16] Huang J., Liang T.J. (1993). A novel hepatitis B virus (HBV) genetic element with Rev response element-like properties that is essential for expression of HBV gene products. Mol. Cell. Biol..

[17] Huang Z.M., Yen T.S. (1994). Hepatitis B virus RNA element that facilitates accumulation of surface gene transcripts in the cytoplasm. J. Virol..

[18] Huang Z.M., Yen T.S. (1995). Role of the hepatitis B virus posttranscriptional regulatory element in export of intronless transcripts. Mol. Cell. Biol..

[19] Zang W.Q., Yen T.S. (1999). Distinct export pathway utilized by the hepatitis B virus posttranscriptional regulatory element. Virology.

[20] Her L.S., Lund E., Dahlberg J.E. (1997). Inhibition of Ran guanosine triphosphatase-dependent nuclear transport by the matrix protein of vesicular stomatitis virus. Science.

[21] Zolotukhin A.S., Felber B.K. (1997). Mutations in the nuclear export signal of human ran-binding protein RanBP1 block the Rev-mediated posttranscriptional regulation of human immunodeficiency virus type 1. J. Biol. Chem..

[22] Zang W.Q., Li B., Huang P.Y., Lai M.M., Yen T.S. (2001). Role of polypyrimidine tract binding protein in the function of the hepatitis B virus posttranscriptional regulatory element. J. Virol..

[23] Li Y., Huang T., Zhang X., Wan T., Hu J., Huang A., Tang H. (2009). Role of glyceraldehyde-3-phosphate dehydrogenase binding to hepatitis B virus posttranscriptional regulatory element in regulating expression of HBV surface antigen. Arch. Virol..

[24] Horke S., Reumann K., Rang A., Heise T. (2002). Molecular characterization of the human La protein·hepatitis B virus RNA.B interaction in vitro.. J. Biol. Chem..

[25] Zhou Z., Sim J., Griffith J., Reed R. (2002). Purification and electron microscopic visualization of functional human spliceosomes. Proc. Natl. Acad. Sci. U.S.A..

[26] Dias A.P., Dufu K., Lei H., Reed R. (2010). A role for TREX components in the release of spliced mRNA from nuclear speckle domains. Nat. Commun..

[27] Zhou Z., Reed R. (2003). Purification of functional RNA-protein complexes using MS2-MBP. Current Protocols in Molecular Biology.

[28] Bennett M., Michaud S., Kingston J., Reed R. (1992). Protein components specifically associated with prespliceosome and spliceosome complexes. Genes Dev..

[29] Folco E.G., Lei H., Hsu J.L., Reed R. (2012). Small-scale nuclear extracts for functional assays of gene-expression machineries. J. Visual. Exp..

[30] Donello J.E., Beeche A.A., Smith G.J., Lucero G.R., Hope T.J. (1996). The hepatitis B virus posttranscriptional regulatory element is composed of two subelements. J. Virol..

[31] Guang S., Felthauser A.M., Mertz J.E. (2005). Binding of hnRNP L to the pre-mRNA processing enhancer of the herpes simplex virus thymidine kinase gene enhances both polyadenylation and nucleocytoplasmic export of intronless mRNAs. Mol. Cell. Biol..

[32] Huang Y., Carmichael G.G. (1996). Role of polyadenylation in nucleocytoplasmic transport of mRNA. Mol. Cell. Biol..

[33] Zhou Z., Licklider L.J., Gygi S.P., Reed R. (2002). Comprehensive proteomic analysis of the human spliceosome. Nature.

[34] Rappsilber J., Ryder U., Lamond A.I., Mann M. (2002). Large-scale proteomic analysis of the human spliceosome. Genome Res..

[35] Jackson B.R., Boyne J.R., Noerenberg M., Taylor A., Hautbergue G.M., Walsh M.J., Wheat R., Blackbourn D.J., Wilson S.A., Whitehouse A. (2011). An interaction between KSHV ORF57 and UIF provides mRNA-adaptor redundancy in herpesvirus intronless mRNA export. PLoS Pathog..

[36] Chen I.H., Li L., Silva L., Sandri-Goldin R.M. (2005). ICP27 recruits Aly/REF but not TAP/NXF1 to herpes simplex virus type 1 transcription sites although TAP/NXF1 is required for ICP27 export. J. Virol..

[37] Koffa M.D., Clements J.B., Izaurralde E., Wadd S., Wilson S.A., Mattaj I.W., Kuersten S. (2001). Herpes simplex virus ICP27 protein provides viral mRNAs with access to the cellular mRNA export pathway. EMBO J..

[38] Zielke B., Thomas M., Giede-Jeppe A., Muller R., Stamminger T. (2010). Characterization of the betaherpesviral pUL69 protein family reveals binding of the cellular mRNA export factor UAP56 as a prerequisite for stimulation of nuclear mRNA export and for efficient viral replication. J. Virol..

[39] Lei H., Zhai B., Yin S., Gygi S., Reed R. (2013). Evidence that a consensus element found in naturally intronless mRNAs promotes mRNA export. Nucleic Acids Res..

[40] Chanarat S., Seizl M., Straber K. (2011). The Prp19 complex is a novel transcription elongation factor required for TREX occupancy at transcribed genes. Genes Dev..

[41] Tange T.O., Shibuya T., Jurica M.S., Moore M.J. (2005). Biochemical analysis of the EJC reveals two new factors and a stable tetrameric protein core. RNA.

[42] Gruber J.J., Zatechka D.S., Sabin L.R., Yong J., Lum J.J., Kong M., Zong W.X., Zhang Z., Lau C.K., Rawlings J. (2009). Ars2 links the nuclear cap-binding complex to RNA interference and cell proliferation. Cell.

[43] Andersen P.R., Domanski M., Kristiansen M.S., Storvall H., Ntini E., Verheggen C., Schein A., Bunkenborg J., Poser I., Hallais M. (2013). The human cap-binding complex is functionally connected to the nuclear RNA exosome. Nat. Struct. Mol. Biol..

[44] Hallais M., Pontvianne F., Andersen P.R., Clerici M., Lener D., Benbahouche Nel H., Gostan T., Vandermoere F., Robert M.C., Cusack S. (2013). CBC-ARS2 stimulates 3’-end maturation of multiple RNA families and favors cap-proximal processing. Nat. Struct. Mol. Biol..

