# Supplementary material for: A Sub-Element in PRE enhances nuclear export of intronless mRNAs by recruiting the TREX complex via ZC3H18
Source: Nucleic Acids Res. 2014 Apr 29;42(11):7305–18. doi: 10.1093/nar/gku350 (PMC4066777; doi:10.1093/nar/gku350)
Supplement: SUPPLEMENTARY DATA [file supp_42_11_7305__index.html]

A Sub-Element in PRE enhances nuclear export of intronless mRNAs by recruiting the TREX complex via ZC3H18 — A Sub-Element in PRE enhances nuclear export of intronless mRNAs by recruiting the TREX complex via ZC3H18 — SUPPLEMENTARY DATA 

# A Sub-Element in PRE enhances nuclear export of intronless mRNAs by recruiting the TREX complex via ZC3H18

## SUPPLEMENTARY DATA

**Files in this Data Supplement:**

- SUPPLEMENTARY DATA
