# Supplementary material for: A Sub-Element in PRE enhances nuclear export of intronless mRNAs by recruiting the TREX complex via ZC3H18
Source: Nucleic Acids Res. 2014 Apr 29;42(11):7305–18. doi: 10.1093/nar/gku350 (PMC4066777; doi:10.1093/nar/gku350)
Supplement: SUPPLEMENTARY DATA [file supp_gku350_nar-00004-a-2014-File009.pdf]

Supplementary Table S1. Targeting sequences of siRNAs and shRNAs.

Target gene	Targeting sequence (5'-3')
ZC3H18 (shRNA)	CCAGCACGGTTCTCATGTAAA
SAFB2 (shRNA)	GCCACCATGTTGTAGCTCAAT
Brr2 (shRNA)	CCTCTCGGCTAATCTCGTA
KIAA1429 (shRNA)	CGGAATATGAAGCAACAAATT
RBM15 (shRNA)	CGCGGAATACAAGACTCTGAA
HNRNPL (shRNA)	CGCTTGAATGTGTTCAAGAAT
RBMX (shRNA)	TGCCCTCTCGTAGAGATGTTT
Acinus (shRNA)	TATCACCACTGAATCACTAAA
Ars2 (shRNA)	CGACCGCAGTGTTAACATT
Negative control (Cntl) (shRNA)	AACAGTCGCGTTTGCTACTTT
TAP (siRNA)	GCGCCATTCGCGAACGATTTT
Thoc2 (siRNA)	GGTTATGCCAAGCTGATTG
Aly (siRNA)	TGGGAAACTGCTGGTGTCCAA
ZC3H18-1 (siRNA)	GCATGAGCTAGACTACGAT
ZC3H18-2 (siRNA)	GGAATGAATTGTAGGTTTA
ZC3H18-3 (siRNA)	GGTCCGATCAGGATTTGGA
Ars2-1 (siRNA)	GAGGAGTGGTTTCGGTCTA
Ars2-2 (siRNA)	CGACCGCAGTGTTAACATT
Ars2-3 (siRNA)	CTACCTGATCGAGGAAGTA
Acinus-1 (siRNA)	GGTCCGTCCTTTCACTTTA
Acinus-2 (siRNA)	GAGGATGAGACAGAGCGTA
Acinus-3 (siRNA)	GAAGGAACCTGAAGCAGAA
Brr2-1(siRNA)	CCTCTCGGCTAATCTCGTA
Brr2-2(siRNA)	GGCTATGCCTACCTCTATA
Brr2-3(siRNA)	GAGCTTATCCGCATGCCAA
Negative control (Cntl) (siRNA)	AACAGTCGCGTTTGCTACTTT

Supplementary Figure S1. PRE enhances cytoplasmic accumulation of the Smad cDNA transcript. (A) Schematic of the Smad reporter constructs. (B) FISH was preformed to determine the nucleocytoplasmic distribution of indicated transcripts 24 hrs after transfection of the reporter plasmids. The FISH probe was same as that used in Figure 1. The cytoplasmic (C) and nuclear (N) fluorescence signal of transfected cells (30 cells per construct) was quantified and the C/N ratios were calculated. Bars

and error bars represent average C/N ratio and standard deviations from three independent experiments.

Supplementary Figure S2. Splicing does not account for PRE-dependent mRNA export. (A) Schematic of the globin reporter constructs. The length of exons, introns and PRE are indicated. The positions of primers are shown as arrows. (B) PCR analysis to assay for cryptic splicing. The β-globin constructs were transfected into HeLa cells. Total RNAs were extracted at 24 hr post-transfection followed by RT-PCRs. Plasmid DNAs were used as PCR templates to generate a size marker for pre-mRNAs (lanes 3, 6 and 9).

Supplementary Figure S3. SiRNA-mediated silencing of ZC3H18 blocks SEP1-mediated mRNA export. (A) HeLa cells were transfected with pcDNA3-ZC3H18 plasmid. 6 hr later, cells were treated with control (Cntl) or ZC3H18 siRNAs. 72 hr later, cells were harvested followed by western analyses to determine the knockdown efficiencies. 10%, 30% and 100% of control knockdown cell lysates were used. The FISH probe was same as that used in Figure 1. (B) HeLa cells were treated with indicated siRNAs. 48 hrs later, the cG-SEP1 plasmid was transfected and FISH was carried out at 24 hr post-transfection to detect the cG-SEP1 mRNA. (C) Similar to (B), but cG-PRE full length construct was transfected.

Supplementary Figure S4. Nucleocytoplasmic distribution of control RNAs used

in Electrophoresis mobility shift assay. DNA sequences of the control RNAs used in EMSA (Figure 4C) were inserted into the 3' of the ORF of cG to make cG-Cntl 1 and cG-Cntl 2 constructs. Both constructs were transfected into HeLa cells and FISH was carried out at 24 hr post-transfection to determine the distribution of indicated mRNAs. The FISH probe was same as that used in Figure 1.

Supplementary Figure S5. SiRNA-mediated silencing of ZC3H18, ARS2, Acinus and Brr2 blocks nuclear export of polyA RNAs. (A) HeLa cells were treated with indicated siRNAs. 72 hrs later, cells were harvested followed by western (ARS2 and Brr2 knockdown) or RT-PCR (Acinus knockdown) analyses to determine the knockdown efficiency. (B) HeLa cells were treated with indicated siRNAs. 72 hrs later, FISH was carried out to examine the distribution of polyA RNAs using the oligo-dT(70) probe.

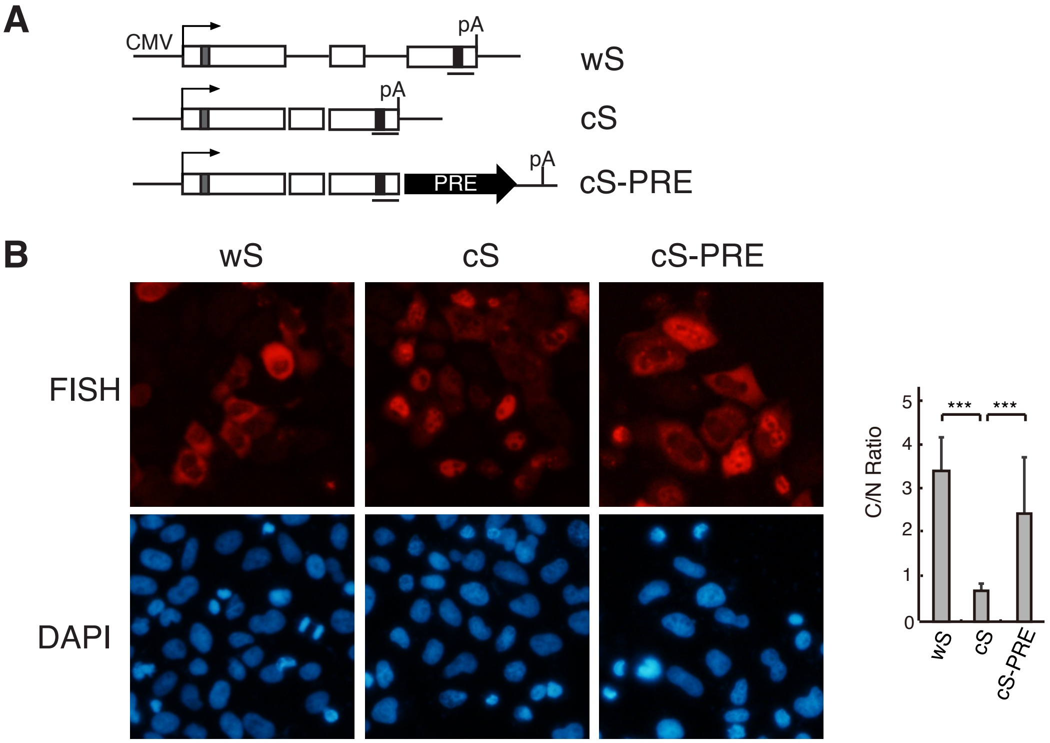
A
CMV
pA
wS
pA
cS
PRE
pA
cS-PRE
B
wS
cS
cS-PRE
FISH
DAPI
C/N Ratio
***
***
0
1
2
3
4
5
wS
cS
cS-PRE

Figure S1

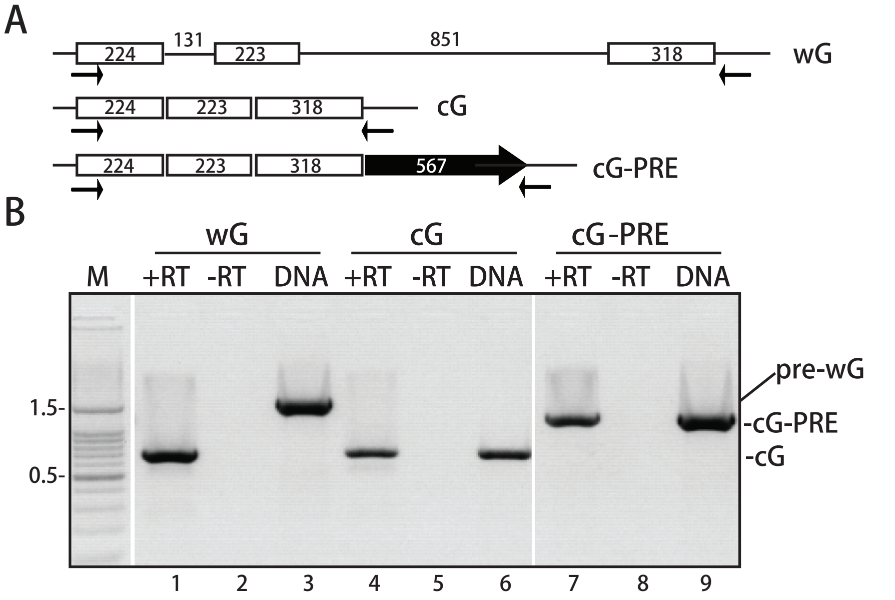
A
131
851
224
223
318
wG
224
223
318
cG
224
223
318
567
cG-PRE
B
wG
cG
cG-PRE
M
+RT
-RT
DNA
+RT
-RT
DNA
+RT
-RT
DNA
1.5-
0.5-
pre-wG
-cG-PRE
-cG
1
2
3
4
5
6
7
8
9

Figure S2

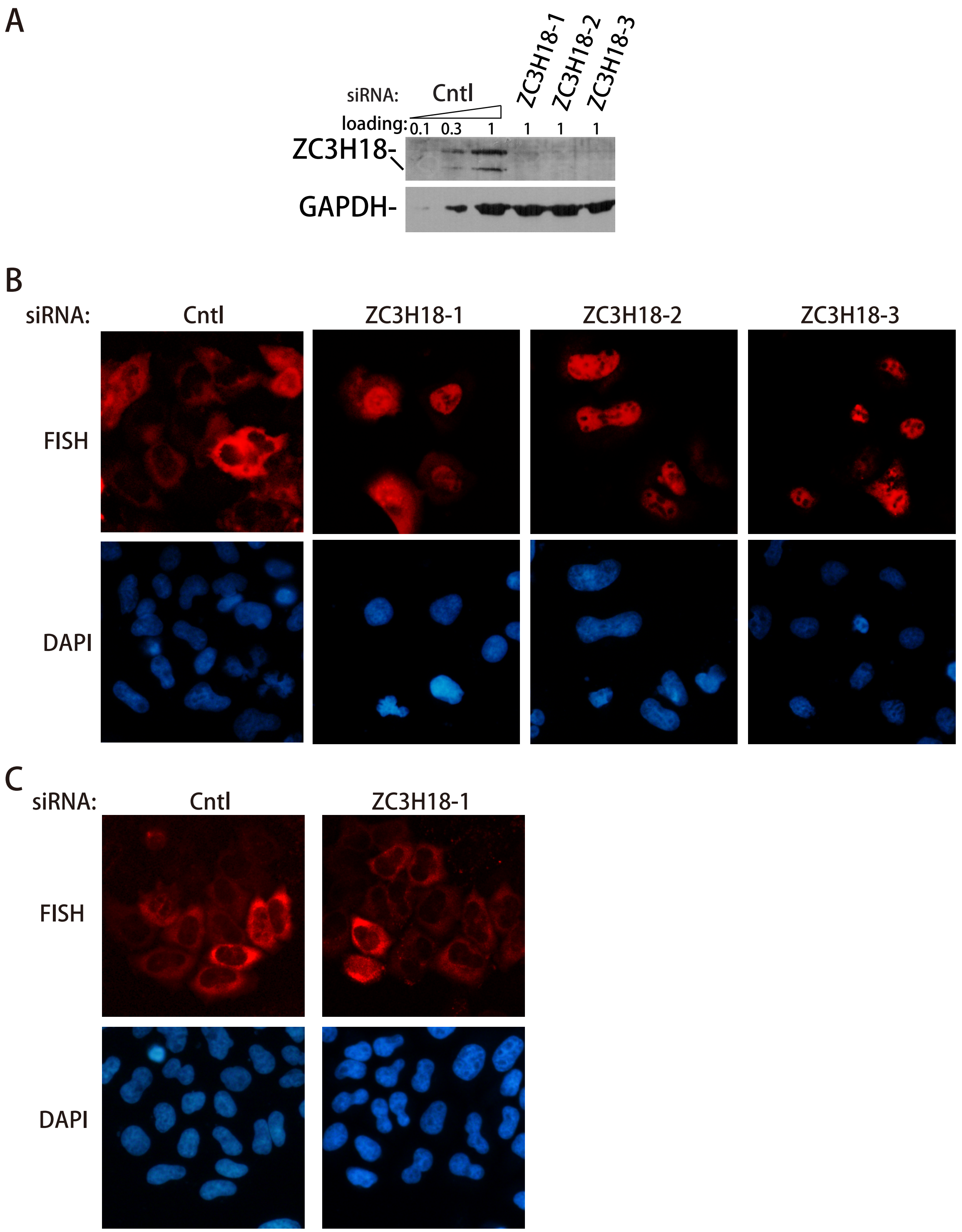
A
siRNA:
Cntl
ZC3H18-1
ZC3H18-2
ZC3H18-3
loading:
0.1
0.3
1
1
1
1
ZC3H18-
GAPDH-
B
siRNA:
Cntl
ZC3H18-1
ZC3H18-2
ZC3H18-3
FISH
DAPI
C
siRNA:
Cntl
ZC3H18-1
FISH
DAPI

Figure S3

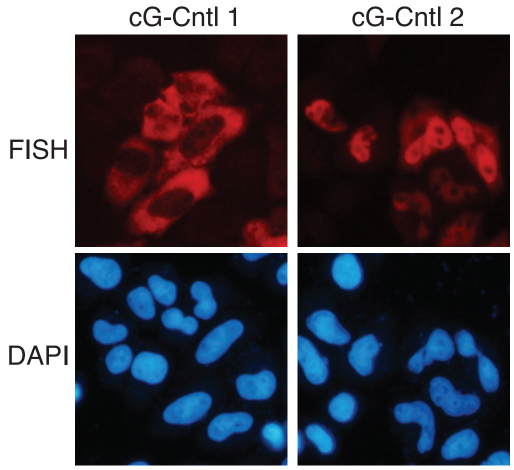

Figure S4

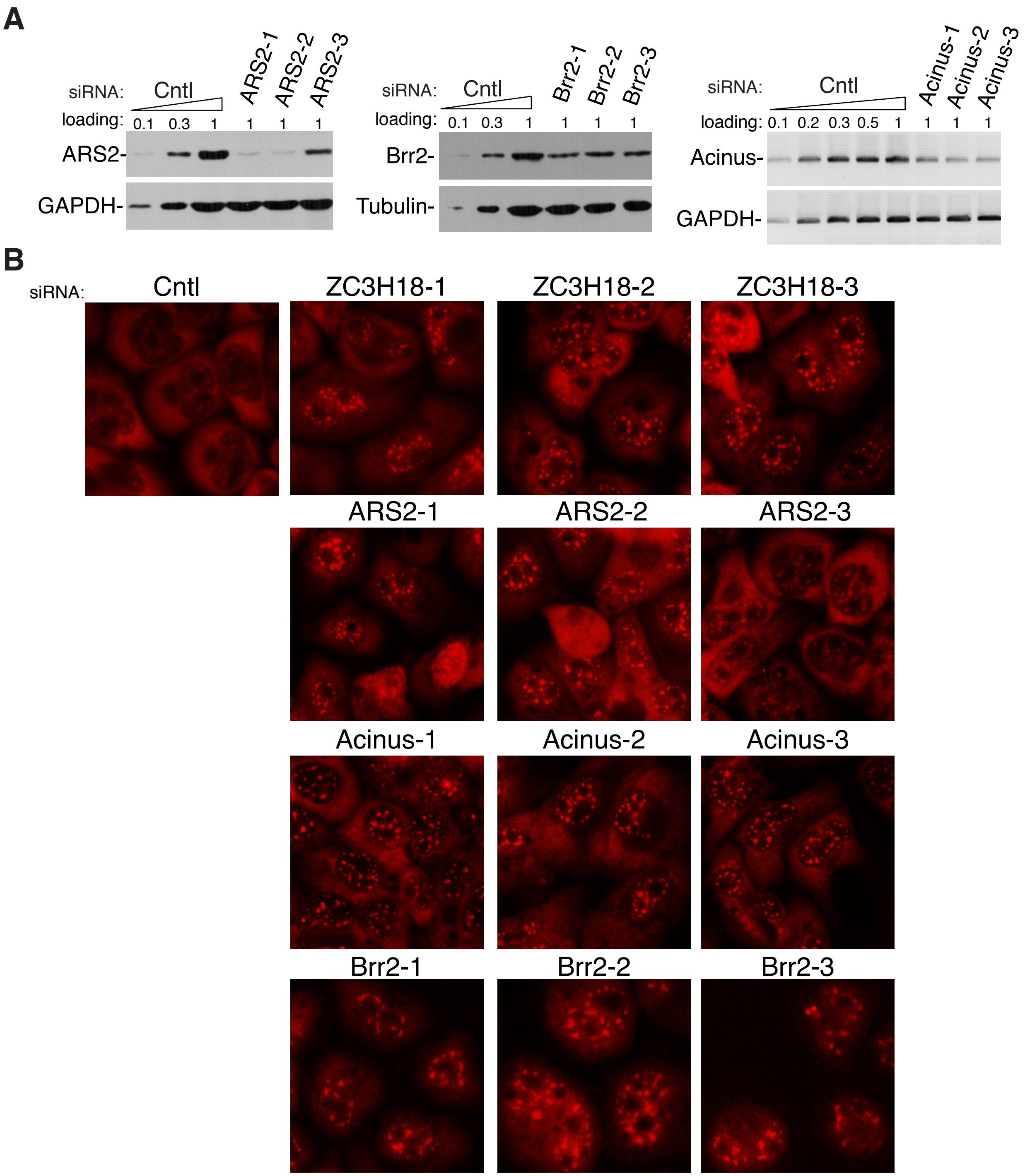
A
siRNA:
Cntl
ARS2-1
ARS2-2
ARS2-3
loading: 0.1 0.3 1 1 1 1
ARS2-
GAPDH-
Brr2-1
Brr2-2
Brr2-3
Brr2-
Tubulin-
Acinus-1
Acinus-2
Acinus-3
loading: 0.1 0.2 0.3 0.5 1 1 1 1
Acinus-
B
ZC3H18-1
ZC3H18-2
ZC3H18-3
Acinus-1
Acinus-2
Acinus-3

Figure S5
